# Dapagliflozin attenuates hepatic steatosis and induces a fasting-mimicking metabolic state in C57BL/6 mice

**DOI:** 10.1007/s00210-026-05723-w

**Published:** 2026-07-23

**Authors:** Pericles Fabrício Gomes-Filho, Carolina da Silva Valentim, Cherley Borba Vieira Andrade, Verônica Aiceles de Medeiros Pinto, Raíssa Leal de Carvalho dos Santos Cunha, Paulo Rogerio Silva de Paiva-Júnior, Rebecca Roosevelt da Conceição Souza Guedes, João Lucas do Céu Amparo, Isalira Peroba Ramos, Luciana Lontro Alves, Aluana Santana Carlos, Jéssica Gomes Pereira, Nathália Medeiros Nehme, Patrícia Cristina Lisboa, Samara Cristina Ferreira-Machado, Kíssila Rabelo, Jorge José de Carvalho, Jemima Fuentes Ribeiro da Silva

**Affiliations:** 1 Laboratory of Ultrastructure and Tissue Biology, Department of Histology and Embryology, State University of Rio de Janeiro, Rio de Janeiro, Brazil; 2 National Center for Structural Biology and Bioimaging, Federal University of Rio de Janeiro, Rio de Janeiro, Brazil; 3 Pre-Clinical Research Laboratory, Iguaçu University, Nova Iguaçu, Rio de Janeiro Brazil; 4 Laboratory of Endocrine Physiology, Department of Physiological Sciences, State University of Rio de Janeiro, Rio de Janeiro, Brazil; 5 Laboratory of Radiological Sciences, Department of Radiological Sciences, State University of Rio de Janeiro, Rio de Janeir, Brazil; 6 Laboratory of Interdisciplinary Medical Research, Oswaldo Cruz Institute, Fiocruz, Rio de Janeiro, Brazil

**Keywords:** MASLD, NT-PGC-1a, Inhibitors SGLT2, High-fat diet, High-fructose, Transmission electron microscopy

## Abstract

Metabolic steatotic liver disease (MASLD) lacks effective pharmacological therapies targeting mitochondrial dysfunction, a central driver of disease progression. We hypothesized that dapagliflozin attenuates MASLD progression by promoting an indirect systemic fasting-mimicking state, thereby proposing the hypothesis of mitochondrial restoring mitochondrial bioenergetics restoration via modulation of the SIRT1–PGC-1α signaling pathway. C57BL/6 mice were fed either a control diet or a high-fat diet, associated, respectively, with drinking water or water with 15% fructose. From the 10th to the 16th week, the animals received, by orogastric gavage, saline solution or dapagliflozin (10 mg/kg). Biochemical analyses, magnetic resonance imaging, oral glucose tolerance tests, stereology, and ultrastructural analysis of the liver, as well as Western blotting using mitochondrial markers, were performed. The study showed that exposure to the high-fat diet associated with fructose effectively mimicked the main aspects of obesity and MASLD. Intervention resulted in a reduction in adiposity without compromising muscle mass, preservation of mitochondrial integrity, improvement in mitochondrial bioenergetics, and attenuation of steatosis and hepatic cellular stress. These results suggest an association with the NT-PGC-1α–NRF1 axis; our study hypothesizes that this acts as a compensatory mechanism. These findings support further investigation of dapagliflozin as a potential therapeutic strategy for MASLD.

**Supplementary Information:**

The online version contains supplementary material available at https://doi.org/10.1007/s00210-026-05723-w.

## Introduction

Obesity and type 2 diabetes mellitus (DM2) have reached epidemic proportions worldwide, largely associated with the excessive consumption of Western diets rich in saturated fats and fructose (Lozano et al. 2016; Li et al. 2020; Litwin and Kulaga 2021). This dysfunctional metabolic scenario promotes an environment of systemic lipotoxicity and glucotoxicity, which are widely recognized as key contributors of the pathogenesis of metabolically related steatotic liver disease (MASLD) (Rorive et al. 2005; Li et al. 2024). This chronic overload of energy substrates in liver tissue can exceed the oxidative capacities of hepatocytes, resulting in intracellular lipid droplets in hepatocytes, hepatic insulin resistance, and oxidative stress, culminating in severe structural alterations in the liver parenchyma (Samuel and Shulman 2016; Sunny et al. 2017).

Liver energy homeostasis is governed by a complex network of metabolic sensors, including AMP-activated protein kinase (AMPK) and NAD + -dependent deacetylase, Sirtuin 1 (SIRT1). These proteins converge to activate peroxisome proliferator-activated receptor coactivator-1-alpha (PGC-1α), a key transcriptional coactivator of mitochondrial biogenesis (Rodgers et al. 2005; Scarpulla 2011). In conditions of obesity and hepatic steatosis, mitochondrial dynamics and homeostasis can be negatively affected, potentially leading to the uncoupling of this pathway. These events may contribute to mitochondrial dysfunction, perpetuating de novo lipogenesis and failure in beta-oxidation of fatty acids, leading to a vicious cycle of deleterious events associated with MASLD (Koliaki et al. 2015; Softic et al. 2016; Sunny et al. 2017; Li et al. 2020).

In this context, dapagliflozin, a sodium-glucose cotransporter 2 (SGLT2) inhibitor, emerges as a promising therapeutic strategy that goes beyond the canonical pathway of glycemic control (Vallon 2015). Although the classic mechanism is the induction of renal glycosuria, recent evidence suggests that these SGLT2 inhibitors promote a systemic state of fasting mimicry. This urinary caloric loss could activate nutrient deprivation sensors, restoring the NAD +/NADH ratio and reactivating the SIRT1/PGC-1α axis (Canto et al. 2010; Packer 2020a, b). However, the expression and stability of this canonical pathway can be negatively affected due to lipotoxicity and glucotoxicity (Zhang et al. 2009). Based on this, alternative pathways, such as the N-terminal truncated variant of PGC-1α (NT-PGC-1α), which retains the ability to coactivate transcription factors such as respiratory nuclear factor 1 (NRF1), may play a crucial and underexplored role in hepatic metabolic recovery through intervention with SGLT2 inhibitors (Chang et al. 2010; Chang et al. 2018; Fernandez-Marcos and Auwerx 2011).

We hypothesize that dapagliflozin promotes indirect systemic fasting-mimicking effects that secondarily lead to hepatic mitochondrial remodeling in MASLD. Our study hypothesizes that the alternative NT-PGC-1α/NRF1 pathway may act as a compensatory mechanism under chronic lipotoxic and glucotoxic conditions.

## Materials and methods

### Animals and experimental design

Approved by CEUA/UERJ (n°010/2024), 3-month-old male C57BL/6 mice were housed under standard controlled conditions. Animals were randomly assigned to a control diet (CTR; AIN-93 M) with pure drinking water or high-fat-fructose diet (HFF; 60% lipids, 26% carbohydrates (fructose free), 14% protein) with 15% (w/v) fructose in the drinking water. From week 10 to 16, animals received daily gavage of vehicle (saline) or 10 mg/kg dapagliflozin (D10), yielding four groups (*n* = 8/group): CTR, CTR + D10, HFF, and HFF + D10.

### Dietary intake, body composition, and metabolic assessments

Water and food intake were monitored per cage during the treatment period. Total energy intake (kJ) was calculated by summing the caloric yields from solid chow (CTR: 16.7 kJ/g; HFF: 23.4 kJ/g) and fructose water (16.7 kJ/g). Consumption values represent the weekly group averages. Weekly body mass was recorded. Body composition (fat and lean mass) was assessed at baseline and post-treatment via magnetic resonance (Minispec LF90, Bruker) to calculate body mass variations (ΔBM), fat mass (ΔFM), and lean mass (ΔLM). After 6 h fast, an OGTT (2 g/kg glucose via gavage) was performed, measuring glycemia from 0 to 120 min to calculate the AUC.

### Euthanasia and biochemical analysis

At week 16, animals were anesthetized (ketamine 240 mg/kg/xylazine 30 mg/kg, i.p.). Blood was collected for serum total cholesterol (TC), HDL, and LDL quantification via enzymatic colorimetry (Bioclin) and insulin levels via ELISA (Cloud-Clone Corp.). HOMA-IR and HOMA-Beta indices were calculated to assess insulin resistance and beta-cell function. Livers were weighed and either fixed (4% paraformaldehyde or 2.5% glutaraldehyde) or snap-frozen at − 80 °C. The variation in sample sizes (*n* = 4–7) for the serum panel was due to pre-analytical technical limitations, specifically insufficient serum volume and mild hemolysis during cardiac puncture procedures.

### Histology, stereology, and ultrasound

Under 1.5% isoflurane anesthesia, ultrasound imaging was performed using a Vevo 2100 (30 MHz). Hepatic samples were processed for H&E and ORO staining. Steatosis volume density (*V*_*V*_) was calculated according to the Delesse principle using STEPanizer software, which accurately reflects the three-dimensional volume of lipid droplets (Mandarim-de-Lacerda and Del Sol 2017). H&E binucleation was estimated by Image-Pro Plus.

### Western blotting and transmission electron microscopy

RIPA-extracted hepatic proteins were subjected to SDS-PAGE and transferred to PVDF membranes. After blocking (5% BSA in TBS-T, 1 h), membranes were probed overnight with primary antibodies (1:1000): AMPK (sc-398861), SIRT1 (1F3), PGC-1α (sc-518025), NRF1 (sc-515360), and β-actin (sc-47778). Following incubation with HRP-conjugated secondary antibody (1:10,000; BA:1050–0.5), bands were quantified using Image Lab. For TEM, liver fragments from all groups were simultaneously post-fixed (osmium tetroxide/potassium ferricyanide), embedded in Poly Bed 812, cut into 70 nm sections, counterstained (uranyl acetate/lead citrate), and imaged at 3000X and 10,000X (JEM 1400FLASH), as previously validated (de Moraes et al. 2021; Ramos et al. 2021; Souza et al. 2022; Neto et al. 2023; Dias et al. 2023).

### Statistical analysis

Data normality and outliers were checked (ROUT/Grubb’s). Multivariate variance was analyzed via PCA and PERMANOVA, alongside Spearman correlation matrices (R v4.5.1). Group comparisons utilized one- or two-way ANOVA with Tukey’s or Bonferroni’s post-hoc tests (GraphPad Prism v10.6.1; *p* < 0.05).

## Results

### Dietary intake and body composition

**Fig. 1:**
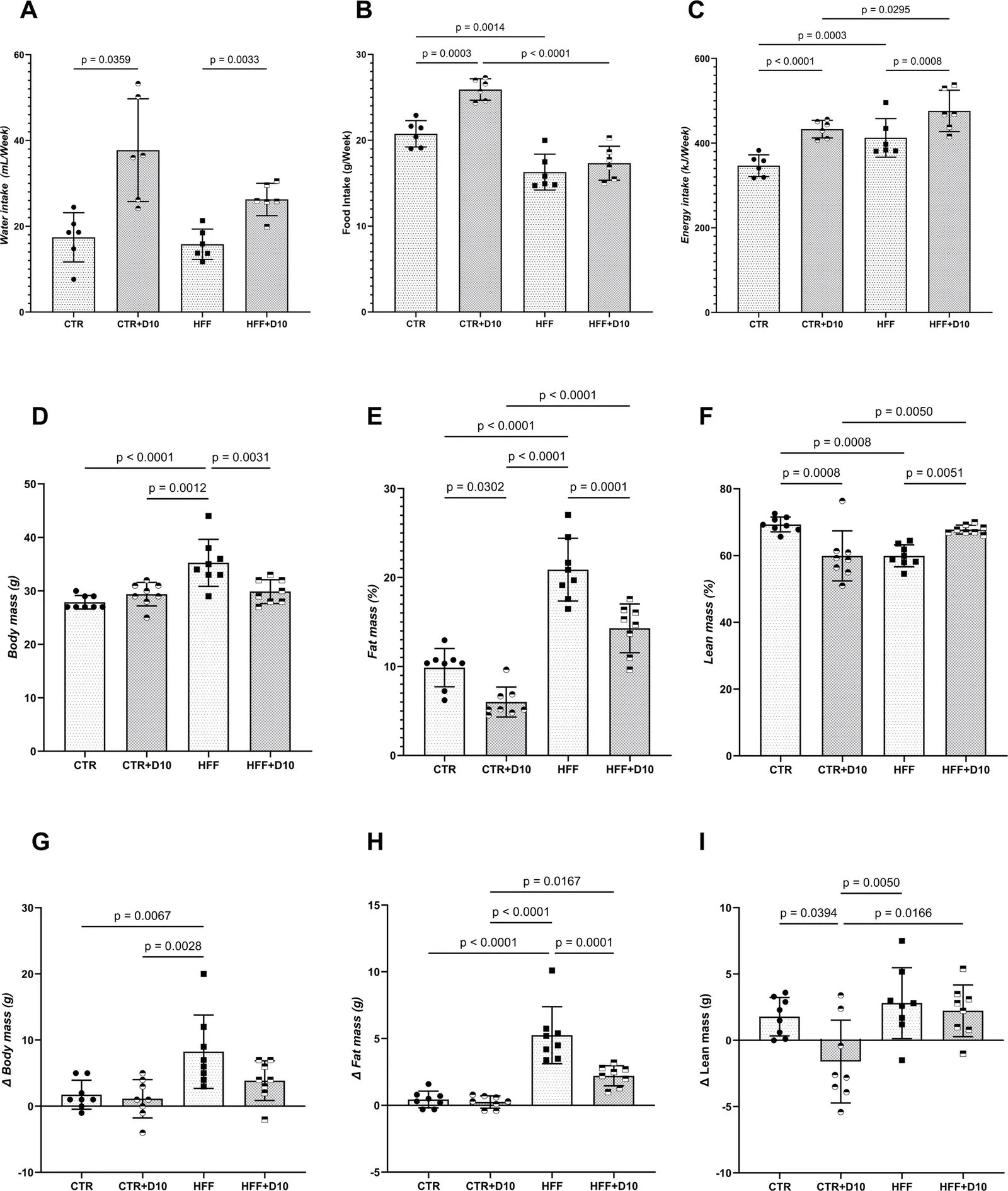
Dietary intake and body composition. **A**–**C** Water intake (mL), food intake (g), and energy intake (kJ). **D–F** Final body mass (g), fat mass (%), and lean mass (%). **G**–**I** Respective variations (*Δ*) in body, fat, and lean mass. Data are mean ± SD. **A**–**C** *n* = 6 weeks/group. **D–I** *n* = 8/group. Statistics: one-way ANOVA (*p* < 0.05). CTR, control; D10, dapagliflozin 10 mg/kg; HFF, high-fat high-fructose diet

Regarding dietary intake, dapagliflozin increased water and food consumption in CTR + D10. In HFF + D10, the drug increased fructose-water intake, which consequently drove a higher total energy consumption compared to HFF, despite similar solid food intake (Fig. 1A–C). The final BM assessment confirmed the HFF diet’s obesogenic effect, significantly increasing weight compared to CTR (+ 26.5%) (Fig. 1D, G). Dapagliflozin counteracted this, reducing BM by 15.25% in the HFF + D10 group compared to HFF. These fluctuations were primarily driven by fat mass (FM), which expanded markedly in HFF (+ 111.6% vs CTR) (Fig. 1E, H). The SGLT2 inhibitor decreased adiposity by 39.1% in CTR + D10 and 31.6% in HFF + D10. Although HFF resulted in a lower relative lean mass (LM) (Fig. 1F), absolute LM accretion was preserved (Fig. 1I). Notably, while the lean-treated group lost lean tissue, HFF + D10 showed no evidence of loss (+ 13.2% vs CTR + D10), demonstrating a protein-sparing effect specifically in the obesogenic context.

### Serum biochemistry profile: metabolic and lipid markers

**Fig. 2:**
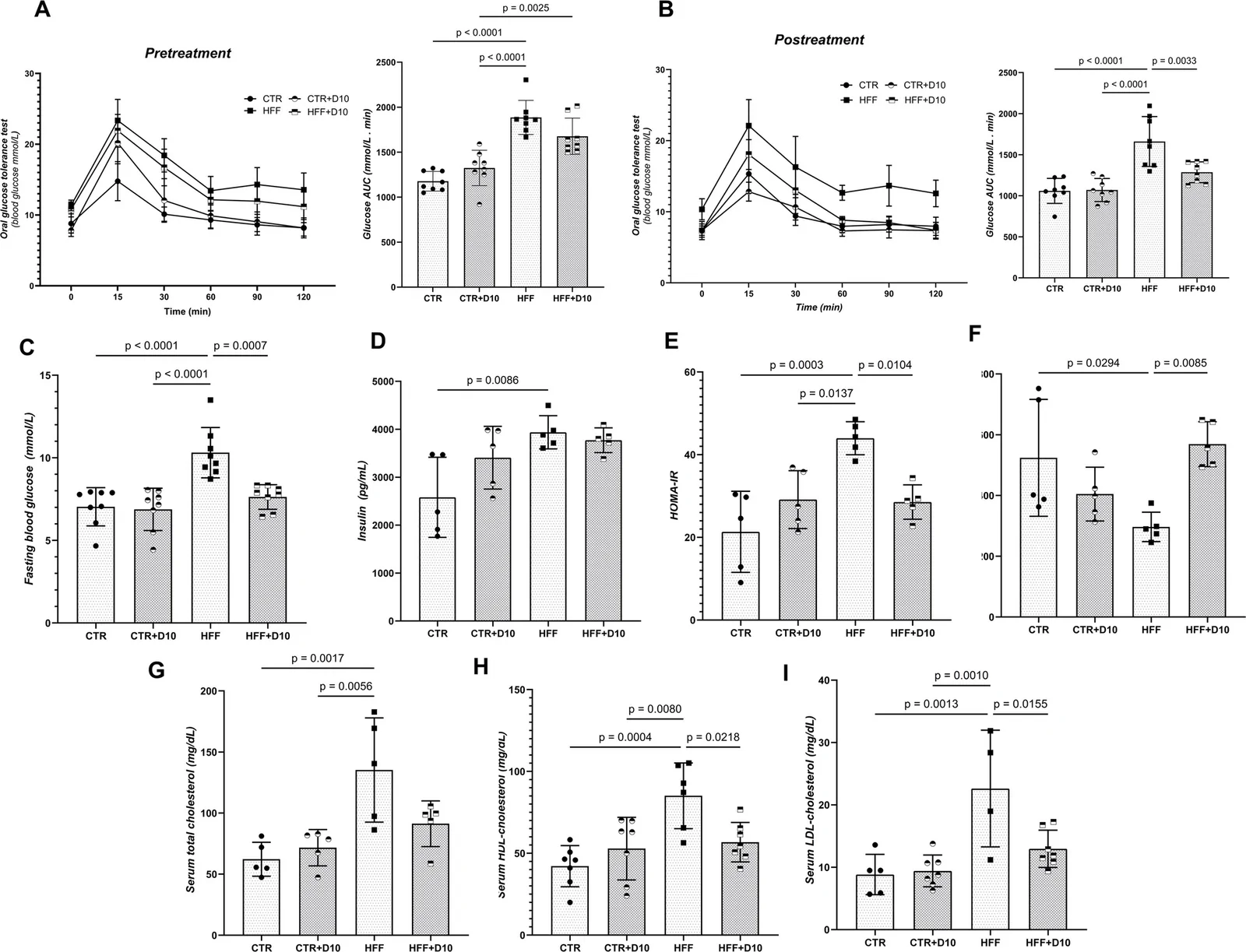
Serum biochemical profile. **A**, **B** OGTT curves and AUC at week 10 (pre-treatment) and week 16 (post-treatment). **C** Final fasting blood glucose. **D** Insulin. **E** HOMA-IR. **F** HOMA-Beta. **G** Total cholesterol (TC), **H** HDL, and **I** LDL. Data are mean ± SD (*N* = 4–8/group). One-way ANOVA (*p* < 0.05)

At week 10, OGTT confirmed HFF-induced glucose intolerance with a larger AUC (+ 60.3% vs CTR) (Fig. 2A). Post-treatment (week 16), dapagliflozin significantly improved systemic glucose handling; while the untreated HFF group maintained severe intolerance (+ 59.7%), HFF + D10 showed a 22.5% reduction in AUC (Fig. 2B). Furthermore, the robust HFF-induced fasting hyperglycemia (+ 46.6% vs CTR) was reversed by dapagliflozin in HFF + D10 (− 26.0% vs HFF), restoring glycemia to lean control levels (Fig. 2C). Consequently, dapagliflozin also significantly attenuated the HFF-induced hyperinsulinemia and improved HOMA-IR and HOMA-Beta indices (Fig. 2D–F). The HFF diet markedly elevated total cholesterol (+ 117.3% vs CTR) (Fig. 2G), alongside a hyperlipoproteinemic profile with increased HDL (+ 102.0%) (Fig. 2H) and LDL (+ 155.9%) (Fig. 2I). In contrast, dapagliflozin marginally reduced total cholesterol in the HFF + D10 group and significantly decreased HDL by 33.3% and LDL by 42.7% compared to the untreated HFF group, restoring lipid parameters toward control values.

### Hepatic histomorphometry, stereological quantification, and ultrasound

**Fig. 3:**
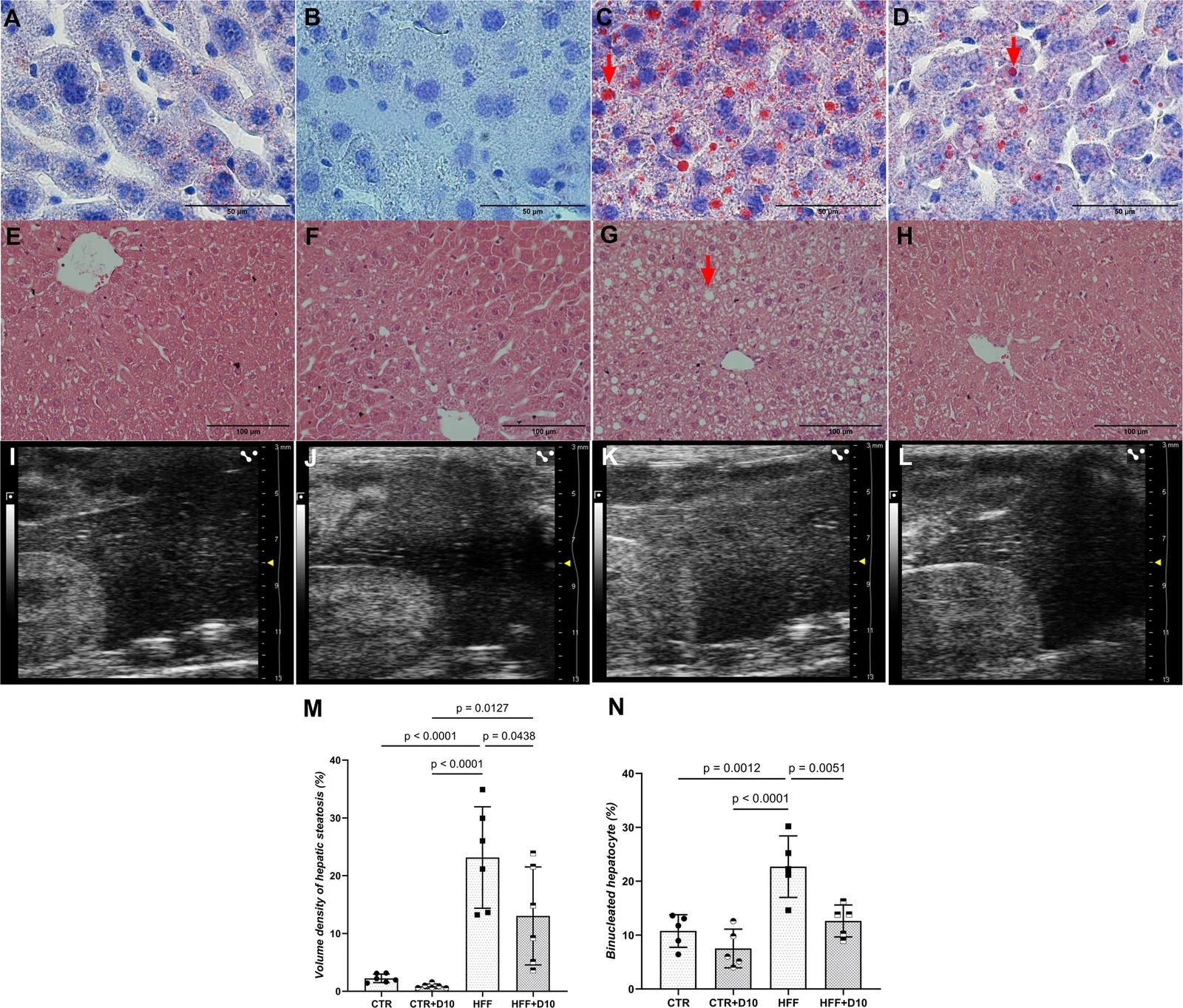
Liver histomorphometry. ORO (**A**–**D**, 50 µm) and H&E (**E–H**, 100 µm) sections: CTR (**A**, **E**), CTR + D10 (**B**, **F**), HFF (**C**, **G**), HFF + D10 (**D**, **H**). Red arrows: steatosis. Steatosis volume density (**M**). Binucleation rate (%) (**N**). Mean ± SD (ORO: *n* = 6; H&E: *n* = 5). One-way ANOVA (*p* < 0.05)

Histological analyses via ORO and H&E staining (Fig. 3A–H) demonstrated negligible lipid deposition in controls, but severe hepatic steatosis and vacuolization in the HFF group. Quantitative *V*_*V*_ analysis confirmed this lipid expansion, while dapagliflozin (HFF + D10) partially reduced steatosis by 43.7% and improved hepatocellular architecture (Figs. 3M). Additionally, the HFF group exhibited a 110.6% increase in hepatocyte binucleation vs CTR, which HFF + D10 decreased by 44.4% (Fig. 3N). Ultrasound corroborated these findings: HFF livers were isoechoic to renal tissue (Fig. 3K), whereas HFF + D10 restored a hypoechoic appearance resembling controls (Fig. 3I, J, L).

### Ultrastructural analysis

**Fig. 4:**
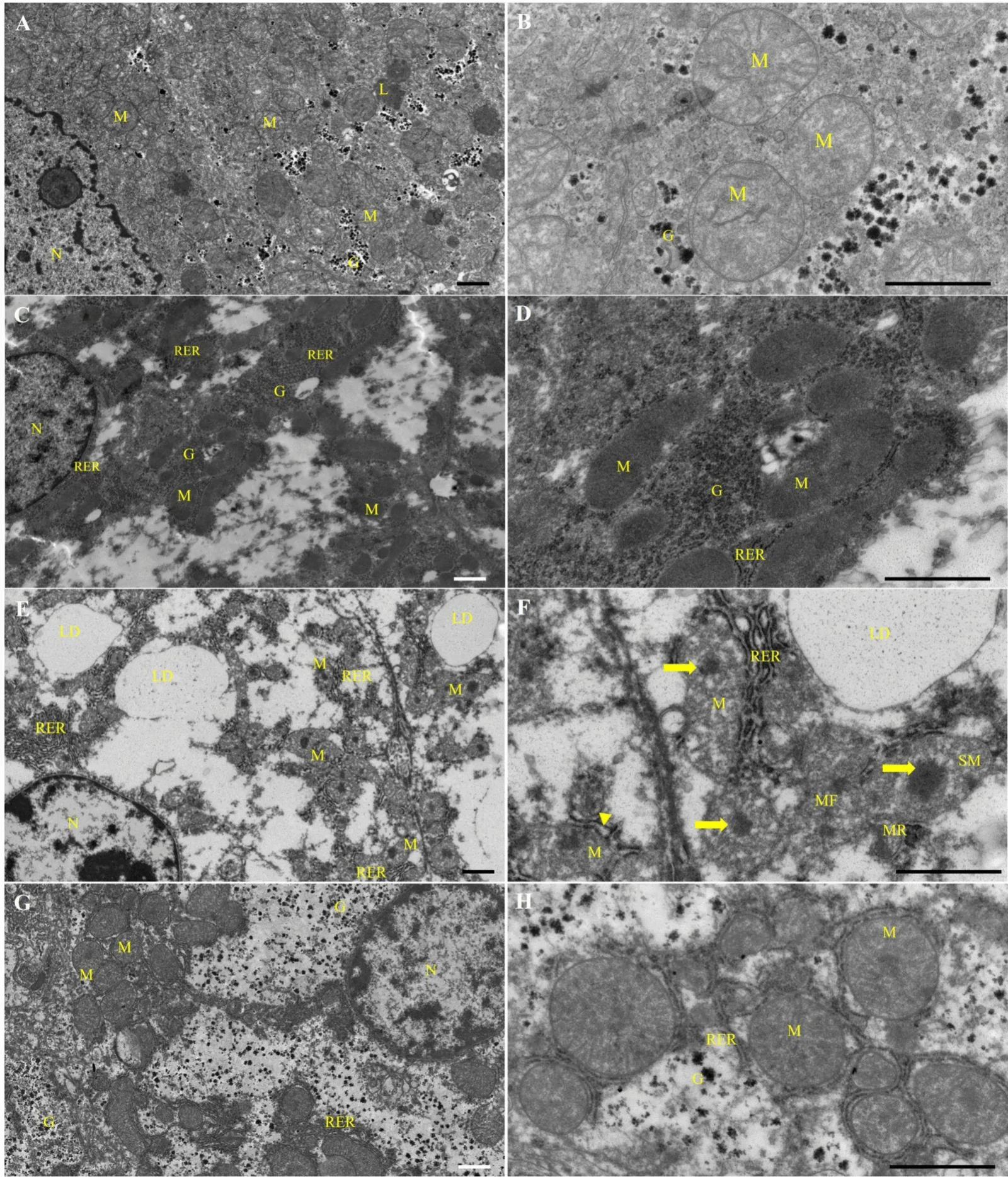
Representative liver TEMs (*N* = 3). CTR (**A**, **B**), CTR + D10 (**C**, **D**), HFF (**E**, **F**), and HFF + D10 (**G**, **H**). Left panels: hepatocytes (3000x; bar = 1 nm). Right panels: mitochondria (10,000x; bar = 1 nm). N, nucleus; RER, rough endoplasmic reticulum; M/MR/MF/SM, normal/ruptured/fusing/swollen mitochondria; G, glycogen; L, lysosomes; LD, lipid droplets. Arrows: ruptured mitochondria or amorphous osmiophilic deposits; arrowheads: dilated RER cisternae

Ultrastructural analysis revealed that CTR hepatocytes exhibited typical euchromatic nuclei, abundant RER, glycogen granules, and normal spherical mitochondria with intact membranes and lamellar cristae (Fig. 4A, B). Conversely, the CTR + D10 group presented decreased organelle density and electron-dense mitochondria with matrix deposits (Fig. 4C, D). The HFF group displayed severe ultrastructural changes, characterized by vesicular cytoplasm, microsteatosis, disorganized RER with dilated cisternae, and scarce glycogen (Fig. 4E). Crucially, HFF mitochondria were dysmorphic, showing ruptured membranes, indistinct cristae, and amorphous osmiophilic deposits (Fig. 4F). Treatment with dapagliflozin (HFF + D10) ameliorated these alterations, restoring organelle density and presenting RER profiles surrounding small mitochondria (Fig. 4G). These hepatocytes showed rounded mitochondria with preserved membranes and elongated cristae, lacking amorphous material, which demonstrates robust mitochondrial restructuring (Fig. 4H).

### Expression profile of mitochondrial biogenesis markers

**Fig. 5:**
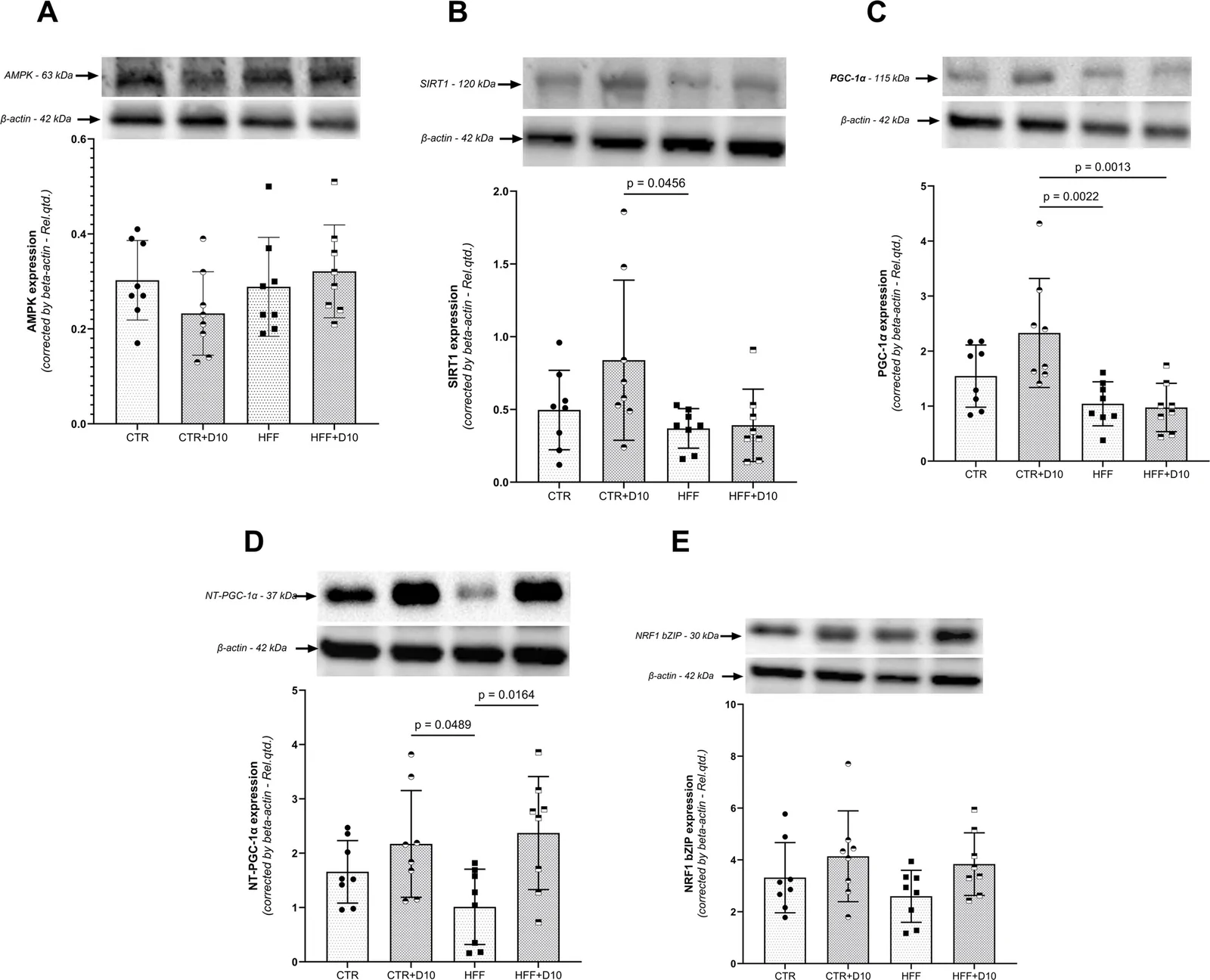
Hepatic mitochondrial biogenesis protein expression. Representative Western blots and densitometry normalized to β-actin: **A** AMPK, **B** SIRT1, **C** PGC-1α, **D** NT-PGC-1α, and **E** NRF1. Mean ± SD (*n* = 8/group). One-way ANOVA (*p* < 0.05)

Mitochondrial biogenesis analysis revealed no significant differences in AMPK (Fig. 5A). However, CTR + D10 increased SIRT1 (+ 126.7% vs HFF; Fig. 5B) and PGC1α (+ 123.5% vs HFF, + 139% vs HFF + D10; Fig. 5C). Furthermore, NT-PGC-1α levels rose in both CTR + D10 (+ 114.4%) and HFF + D10 (+ 134.2%) compared to HFF (Fig. 5D). NRF1 displayed a similar non-significant increasing trend in treated groups (Fig. 5E).

### General correlation matrix (metabolic and molecular interactions) and hybrid correlation matrix (metabolic rewiring and intra-group associations) and multivariate analysis of metabolic and molecular profile

**Fig. 6:**
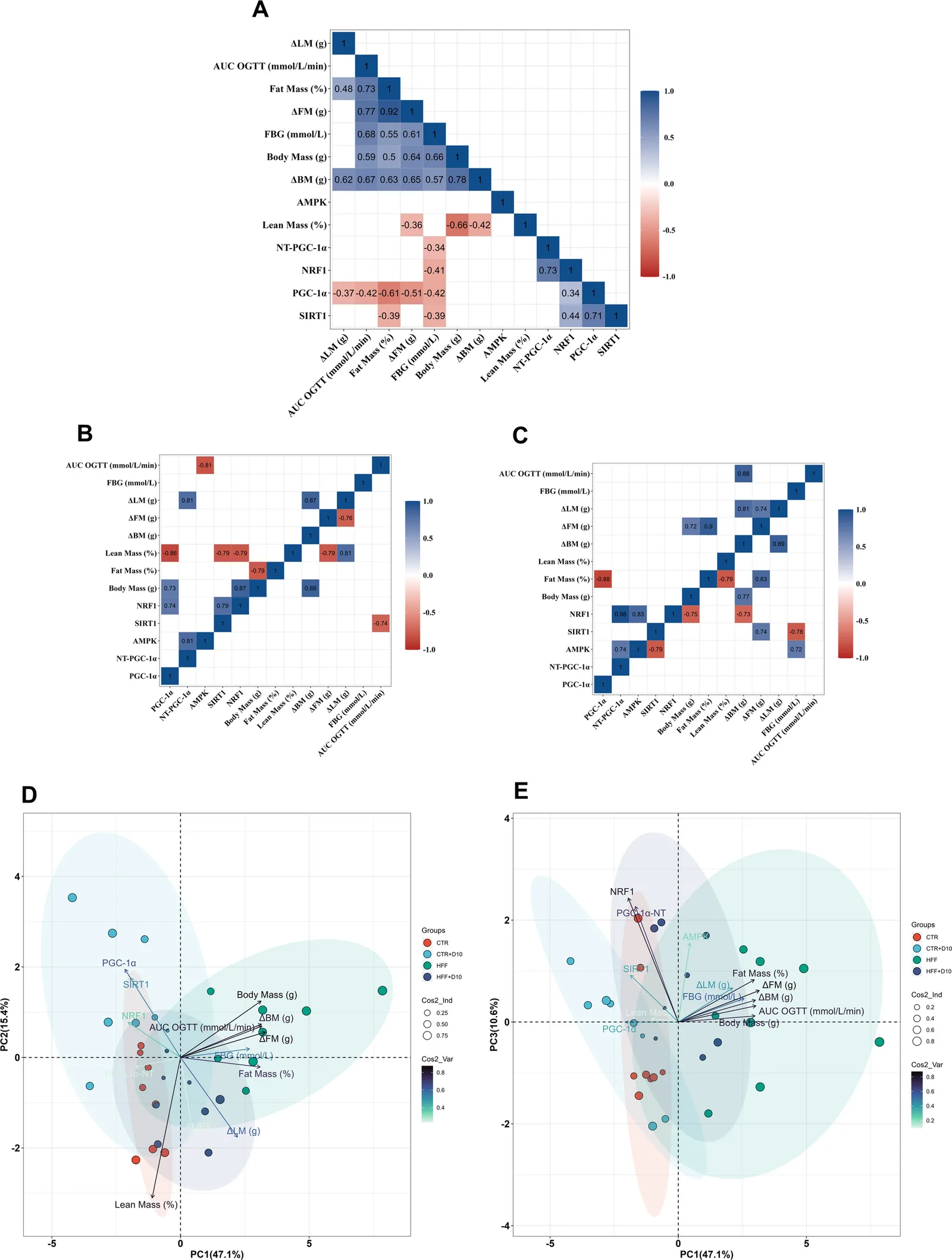
Spearman correlation matrices of metabolic and molecular parameters. **A** General matrix (rs) (n = 32). Blue and red indicate positive (rs > 0) and negative (rs < 0) correlations, respectively; blank cells denote non-significance (*p* > 0.05). Variables are hierarchically clustered. Hybrid matrices contrast intra-group profiles (*n* = 8/group): **B** CTR (upper) vs CTR + D10 (lower); **C** HFF (upper) vs HFF + D10 (lower). PCA of metabolic and molecular profiles. **D** PC1 vs PC2 biplot (62.5% cumulative variance). **E** PC1 vs PC3 projection (10.6% variance). Ellipses: 95% confidence intervals. Color gradient and point size: representation quality (Cos.^2^). Groups (*n* = 8/group): CTR (red), CTR + D10 (light blue), HFF (green), and HFF + D10 (dark blue)

Global correlation analysis (Fig. 6A) showed metabolic burden inversely relates to molecular adaptation. BM and adiposity positively correlated with fasting glucose (rs = 0.66, rs = 0.55) and FM% (rs = 0.50). LM% negatively correlated with ΔFM (rs =  − 0.36) and BM (rs =  − 0.66). Obesogenic markers suppressed PGC-1α and SIRT1 negative correlations with fat% and fasting glucose (rs: − 0.39 and − 0.61). Notably, NT-PGC-1α strongly correlated with NRF1 (rs = 0.73).

Hybrid matrices (Fig. 6) revealed treatment shifts. In CTR (Fig. 6B, upper), growth coupled with NRF1 (rs = 0.87) and PGC-1α (rs = 0.73), while AMPK inversely correlated with AUC OGTT (rs =  − 0.81). In CTR + D10 (lower), SIRT1 negatively regulated AUC OGTT (rs =  − 0.74) and ΔBM (rs =  − 0.71). In HFF (Fig. 6C, upper), adiposity correlated with AUC OGTT (rs = 0.88), ΔLM (rs = 0.74), and suppressed PGC-1α (rs =  − 0.88); NRF1 correlated with NT-PGC-1α (rs = 0.98). HFF + D10 (lower) uncoupled adiposity from glucose tolerance; SIRT1 inversely associated with fasting glucose (rs =  − 0.78) and AMPK (rs =  − 0.79), and NRF1 with BM (rs =  − 0.75).

PCA cumulatively explained 73.1% of the data variance (PC1: 47.1%, PC2: 15.4%, PC3: 10.6%). In the PC1 vs PC2 projection (Fig. 6D), the HFF group segregated from controls, driven by adiposity and glycemic markers, while controls clustered with PGC-1α, SIRT1, and LM%. The PC1 vs PC3 projection (Fig. 6E) revealed HFF + D10 shifting toward NRF1 and NT-PGC-1α. PERMANOVA confirmed distinct metabolic signatures among groups (${F}_{\mathrm{3,28}}$ = 16.72, total *R*^2^ = 0.6418). Pairwise comparisons showed HFF completely diverged from CTR (*R*^2^ = 0.6418). Crucially, dapagliflozin significantly remodeled the HFF + D10 profile compared to HFF (*R*^2^ = 0.4250), whereas CTR and CTR + D10 remained statistically similar (*R*^2^ = 0.003).

## Discussion

This investigation elucidates how SGLT2 inhibition remodels hepatic metabolism in obesity, hypothesizing a compensatory mechanism via the NT-PGC-1α/NRF1 pathway. PCA segregated the HFF group due to adiposity and glycemic dysregulation, confirming the robustness of the lipotoxicity model (Lozano et al. 2016; Li et al. 2020). Interestingly, dapagliflozin promoted a metabolic dissociation. Beyond the canonical effects of glycosuria, the PC1 versus PC3 projection suggests adaptive modulation driven by NRF1 and the NT-PGC-1α variant (Hawley et al. 2016; Packer 2020b; Jhuo et al. 2024).

Correlation matrices mapped the functional architecture of these alterations. In HFF, adiposity strongly correlated with glycemia (Rorive et al. 2005; Litwin and Kulaga 2021), actively suppressing the SIRT1/PGC-1α bioenergetic axis (Sunny et al. 2017). Conversely, the CTR group maintained a homeostatic AMPK/SIRT1/PGC-1α/NRF1 pathway (Rodgers et al. 2005). Crucially, in CTR + D10, SIRT1 emerged as the primary driver of glycemic homeostasis, statistically supporting the SGLT2 inhibitor-induced fasting mimicry hypothesis (Ferrannini et al. 2014; Packer 2020a, b). In the HFF group, lipotoxicity drove mitochondrial suppression (Messier et al. 2007; Koliaki et al. 2015). Furthermore, HFF + D10 disrupted the obesogenic pattern by decoupling glycemia from adiposity, while NRF1 negatively correlated with BM, indicating drug-induced mitochondrial modulation (Rodgers et al. 2005; Ferrannini et al. 2014; Li et al. 2024). The negative AMPK/SIRT1 relationship in treated mice requires further investigation (Canto et al. 2010).

Increased water and food consumption in treated groups reflects osmotic diuresis and compensatory hyperphagia driven by glycosuria. Notably, dapagliflozin decoupled energy intake from body mass in HFF + D10, demonstrating that the urinary caloric deficit overrides compensatory ingestion, promoting weight loss despite the hypercaloric supply (Devenny et al. 2012). Body composition revealed that CTR + D10 lost both fat and lean mass. This suggests that fasting mimicry may recruit amino acids for gluconeogenesis in subjects with low lipid reserves (Vallon 2015; Packer 2020b). HFF induced severe fat accumulation (Messier et al. 2007). However, HFF + D10 decoupled weight variation from adiposity; despite reductions in body mass and adiposity, lean mass was uniquely protected compared to CTR + D10, indicating a protein-sparing effect under therapeutic glycosuria (Bolinder et al. 2014; Sasaki et al. 2019).

Pre-treatment OGTT confirmed HFF-induced insulin desensitization (Samuel and Shulman 2016). Post-treatment, HFF + D10 improved glucose metabolism and insulin resistance (HOMA-IR) entirely via indirect systemic effects. As SGLT2 is absent in pancreatic cells and its inhibition does not directly affect insulin secretion (Chae et al. 2020), the hepatic improvements are strictly secondary to the systemic fasting-mimicking state driven by renal glycosuria (Packer 2020a, b). This normoglycemic restoration suggests dapagliflozin breaks the lipotoxicity cycle, allowing the liver to recover endogenous glucose production (Kahn et al. 2006). The lipid profile confirmed the normalization of TC, HDL, and LDL in HFF + D10, corroborating the lipid-lowering efficacy of dapagliflozin (Calapkulu et al. 2019; Khanna et al. 2025).

Stereological analysis provided structural evidence of therapeutic efficacy. The reduction in steatosis VV in HFF + D10 demonstrates the drug’s potency in attenuating ectopic triglyceride accumulation, possibly via improved mitochondrial beta-oxidation (Mandarim-de-Lacerda 2003; Yamaguchi et al. 2007). Additionally, the significant reduction in hepatocyte binucleation indicates attenuated lipotoxic genetic damage (Thongnak et al. 2020).

Ultrastructurally, CTR + D10 exhibited swollen mitochondria and increased glycogen, suggesting a fasting state. Conversely, HFF mitochondria showed severe damage and amorphous osmiophilic deposits. In HFF + D10, mitochondria were preserved, and glycogen deposits suggested maintenance of metabolic fasting (Duranova et al. 2020; Talari et al. 2025). These analyses corroborate the molecular data presented in this study.

Molecularly, dapagliflozin triggered PGC-1α isoform switching. While CTR + D10 canonically activated the full-length SIRT1/PGC-1α pathway, consistent with classic fasting mimicry (Canto et al. 2010; Packer 2020a; Rodgers et al. 2005), HFF suppressed the classical PGC-1α protein. The key finding is the selective induction of the truncated, degradation-resistant NT-PGC-1α variant in treated obese mice (Zhang et al. 2009; Chang et al. 2010; Chang et al. 2018; Fernandez-Marcos and Auwerx 2011). The strong NT-PGC-1α/NRF1 correlation suggests an association between dapagliflozin treatment and the mitigation of hepatic lipotoxicity. These findings hypothesize that this alternative axis may act as a compensatory mechanism to restore hepatic bioenergetics, successfully dissociating steatosis from mitochondrial dysfunction through adiposity-independent transcriptional mechanisms (Scarpulla 2011; Thongnak et al. 2020), although further functional studies are required to confirm this mechanistic pathway.

This study has limitations. First, only total AMPK was measured, precluding conclusions about direct pathway activation via Thr172 phosphorylation; thus, any modulation is hypothesized based on downstream targets. Furthermore, the absence of evaluations for mitochondrial dynamics markers, such MFN2 and DRP1, and biogenesis markers such as NRF-2 and TFAM represents additional limitations. Triglyceride levels were not analyzed, although they are considered an important indicator of metabolic risk in obesity. Finally, although evidence suggests a possible role for the NT-PGC-1α isoform in metabolic adaptation, there is still no robust data to fully support its mechanistic involvement; therefore, further studies are needed to elucidate this pathway.

## Conclusions

The HFF diet successfully induced MASLD and mitochondrial suppression. Dapagliflozin promoted systemic and hepatic remodeling, reducing adiposity and steatosis while preserving LM and mitochondrial integrity. Crucially, treatment uncoupled adiposity from liver dysfunction and suggests the recruitment of an alternative fasting-mimicking rescue pathway via the NT-PGC-1α-NRF1 axis. This isoform switching is hypothesized to restore bioenergetics, highlighting dapagliflozin as a potent therapeutic strategy against MASLD beyond glycemic control.

## Supplementary Information

Below is the link to the electronic supplementary material.

ESM 1 (PDF 37.4 MB)

## Data Availability

All source data for this work (or generated in this study) are available upon reasonable request.
