# Supplementary material for: Dapagliflozin attenuates hepatic steatosis and induces a fasting-mimicking metabolic state in C57BL/6 mice
Source: Naunyn Schmiedebergs Arch Pharmacol. 2026 Jul 23;399(13):21509–20. doi: 10.1007/s00210-026-05723-w (PMC13695597; doi:10.1007/s00210-026-05723-w)

AMPK

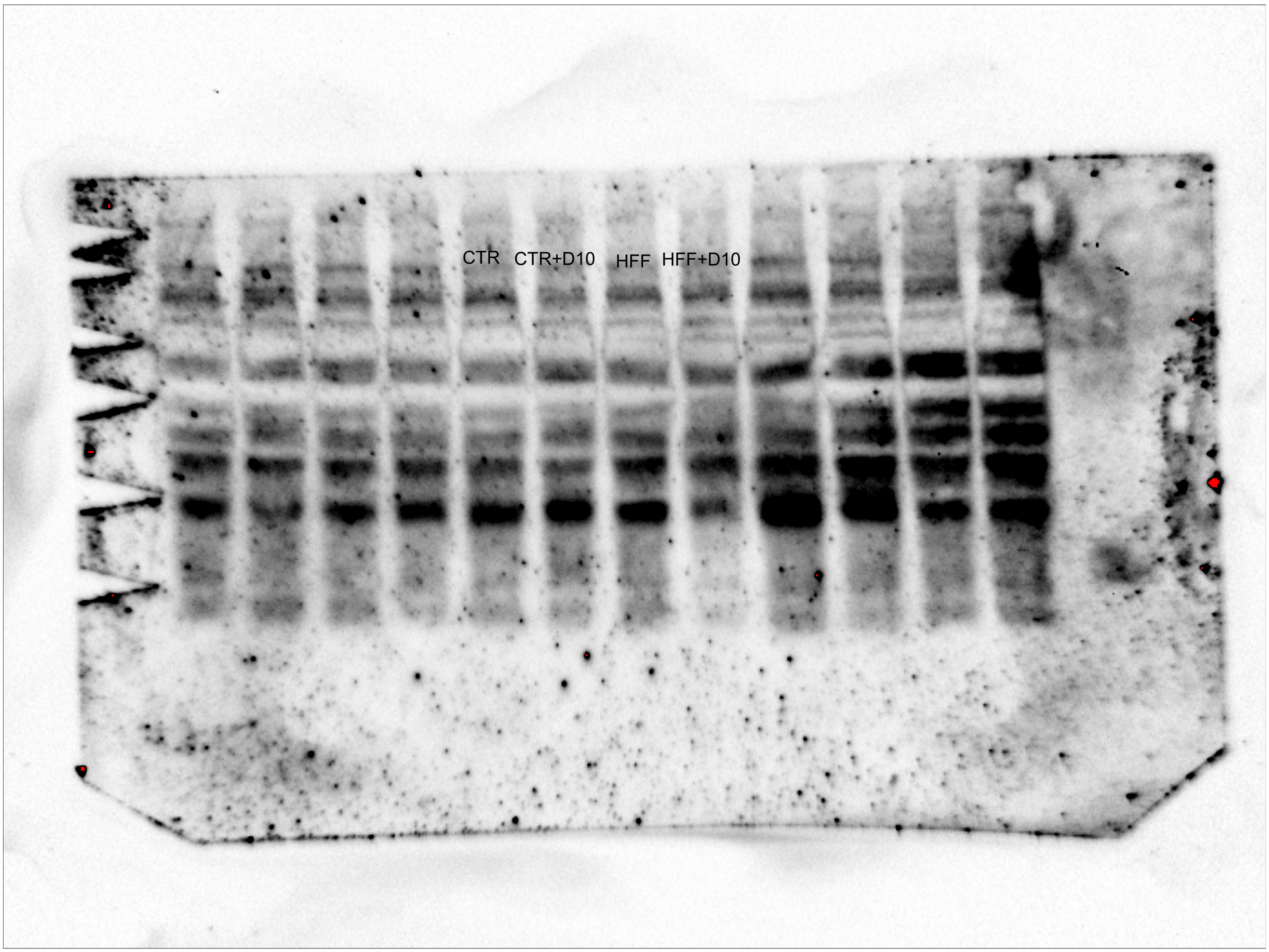

DFP 2025-10-21_Beta actin_ AMPKec

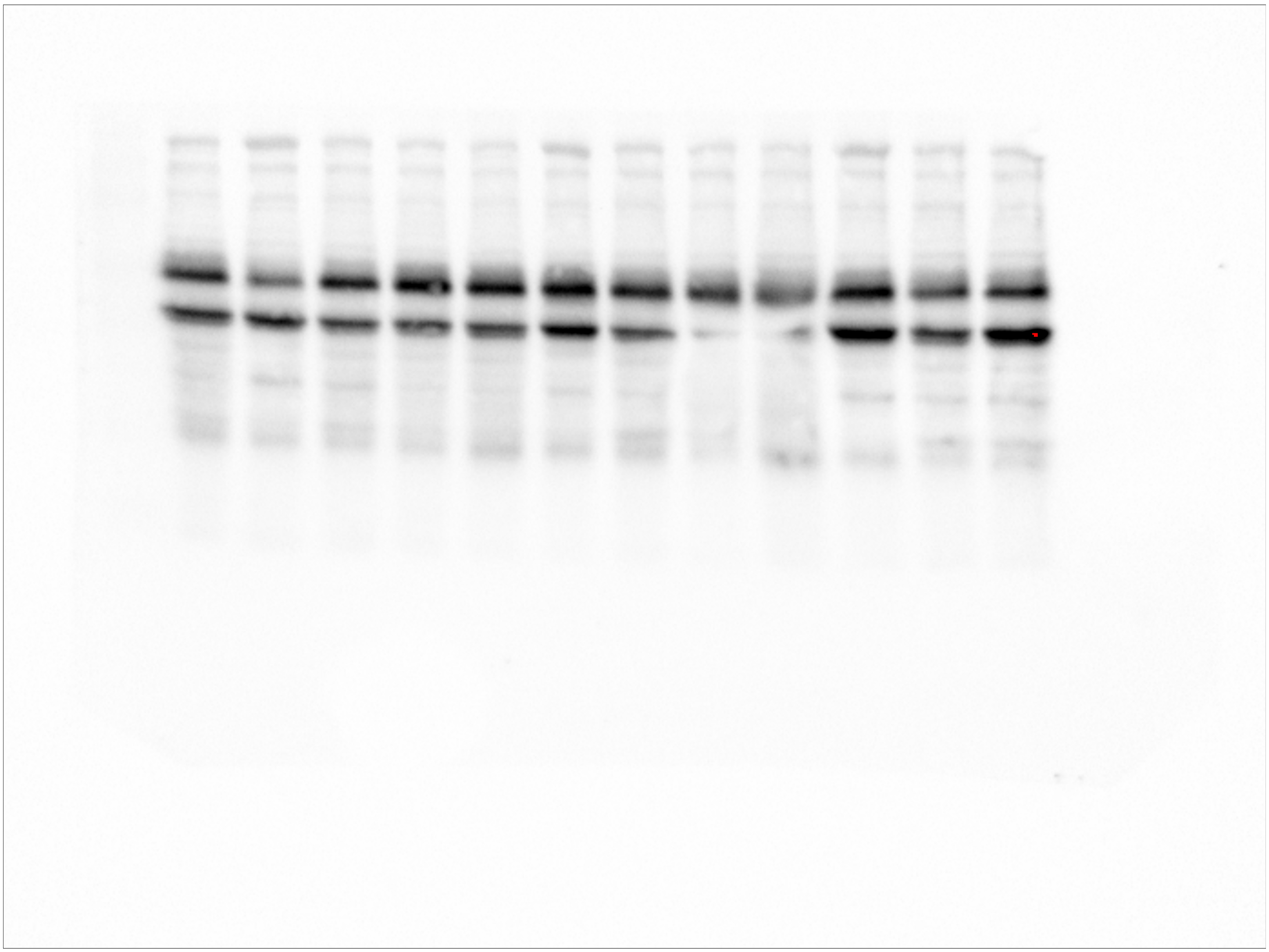

Beta actin_NRF1

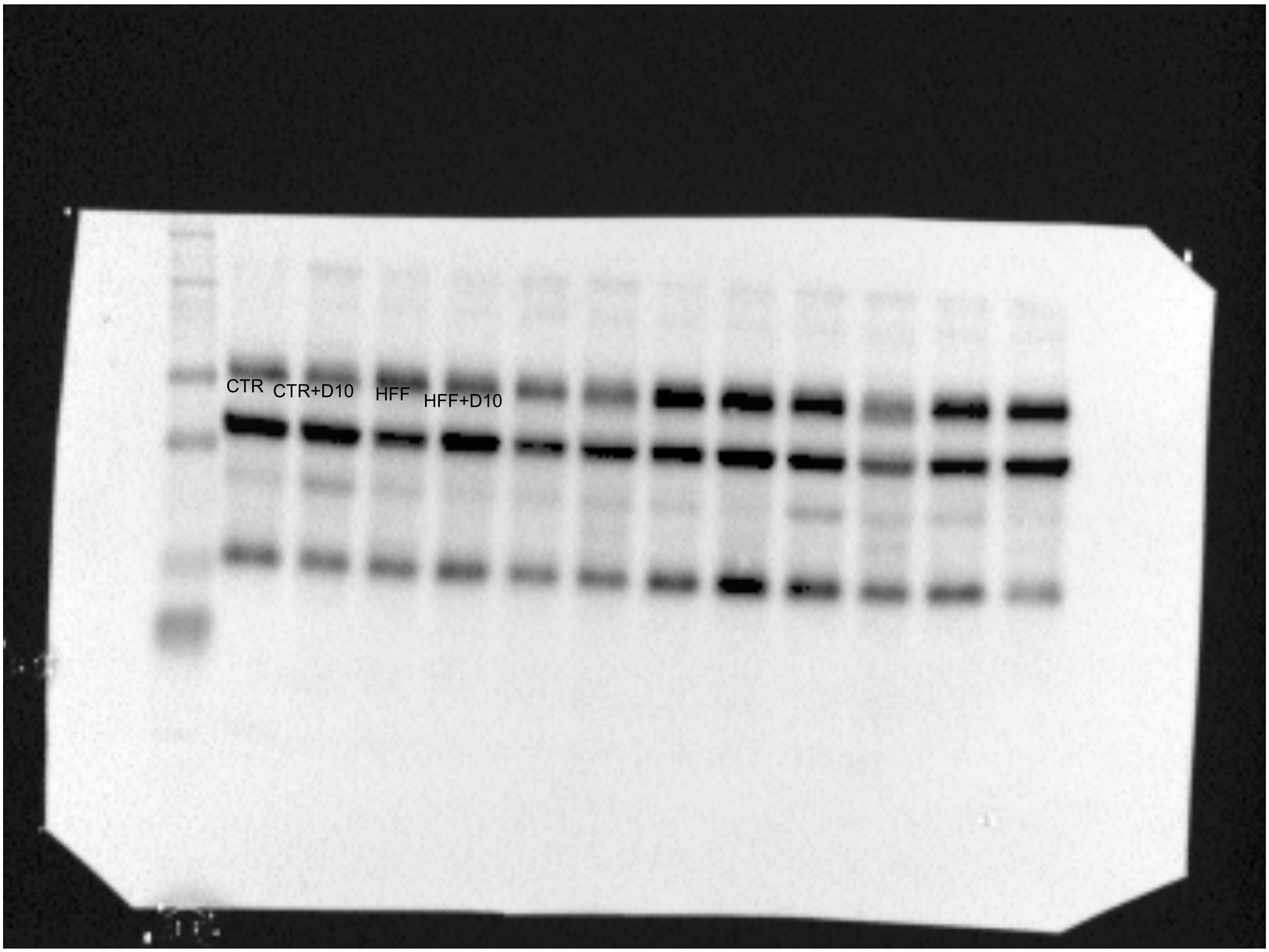

Beta actin_NT-PGC1a

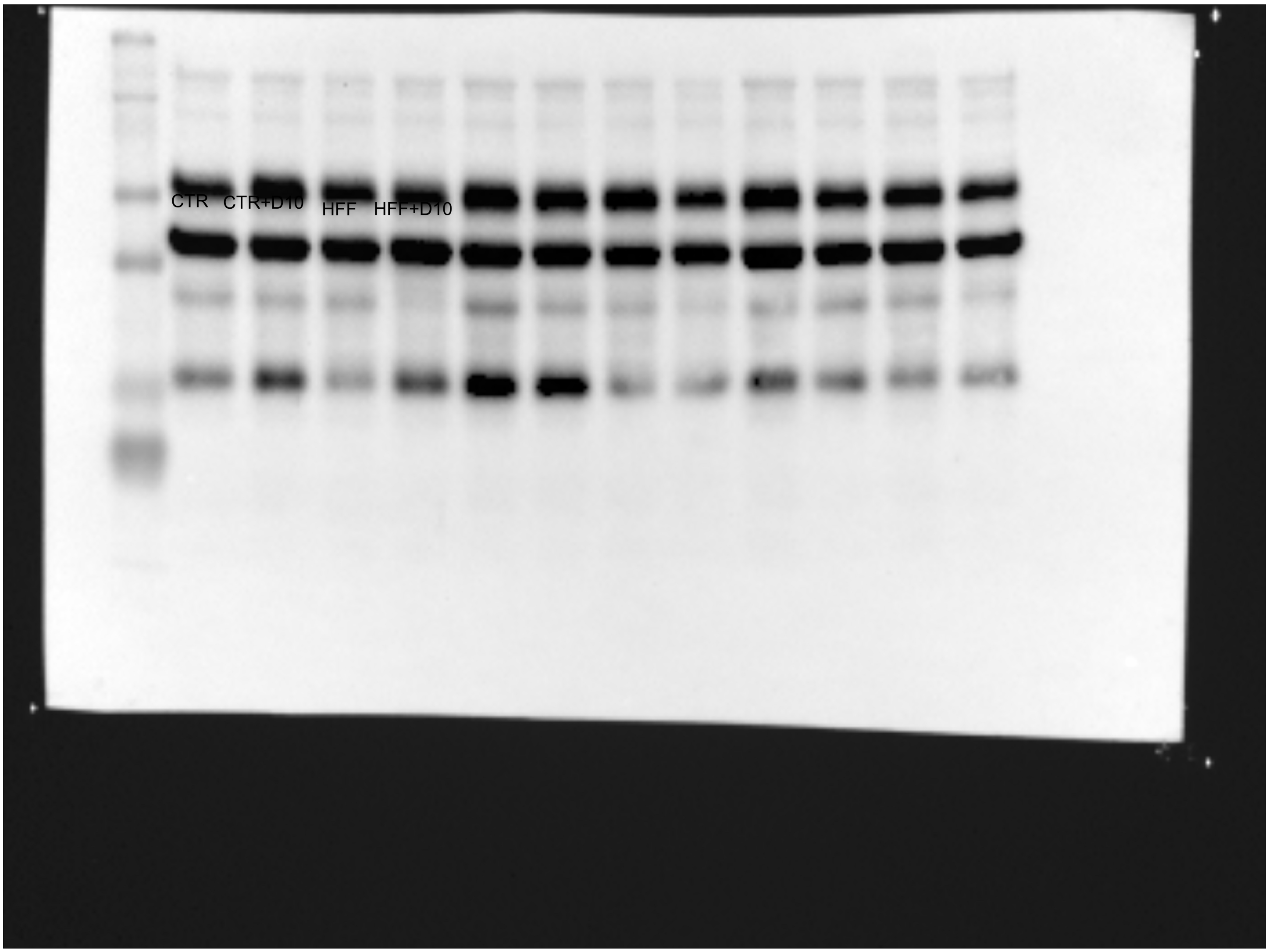

Beta actin_PGC1a

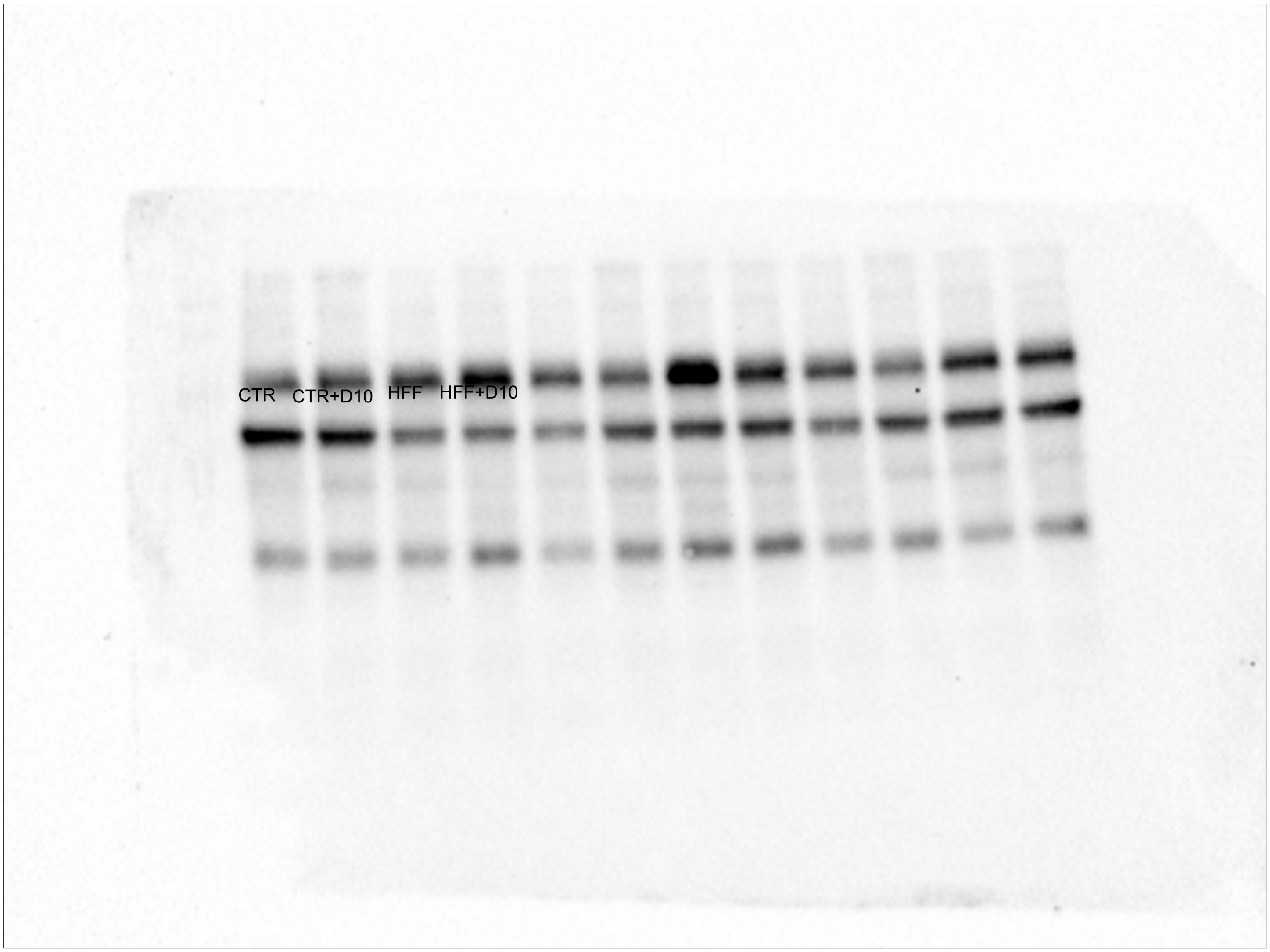

Membrana_Beta actin_SIRT1sec

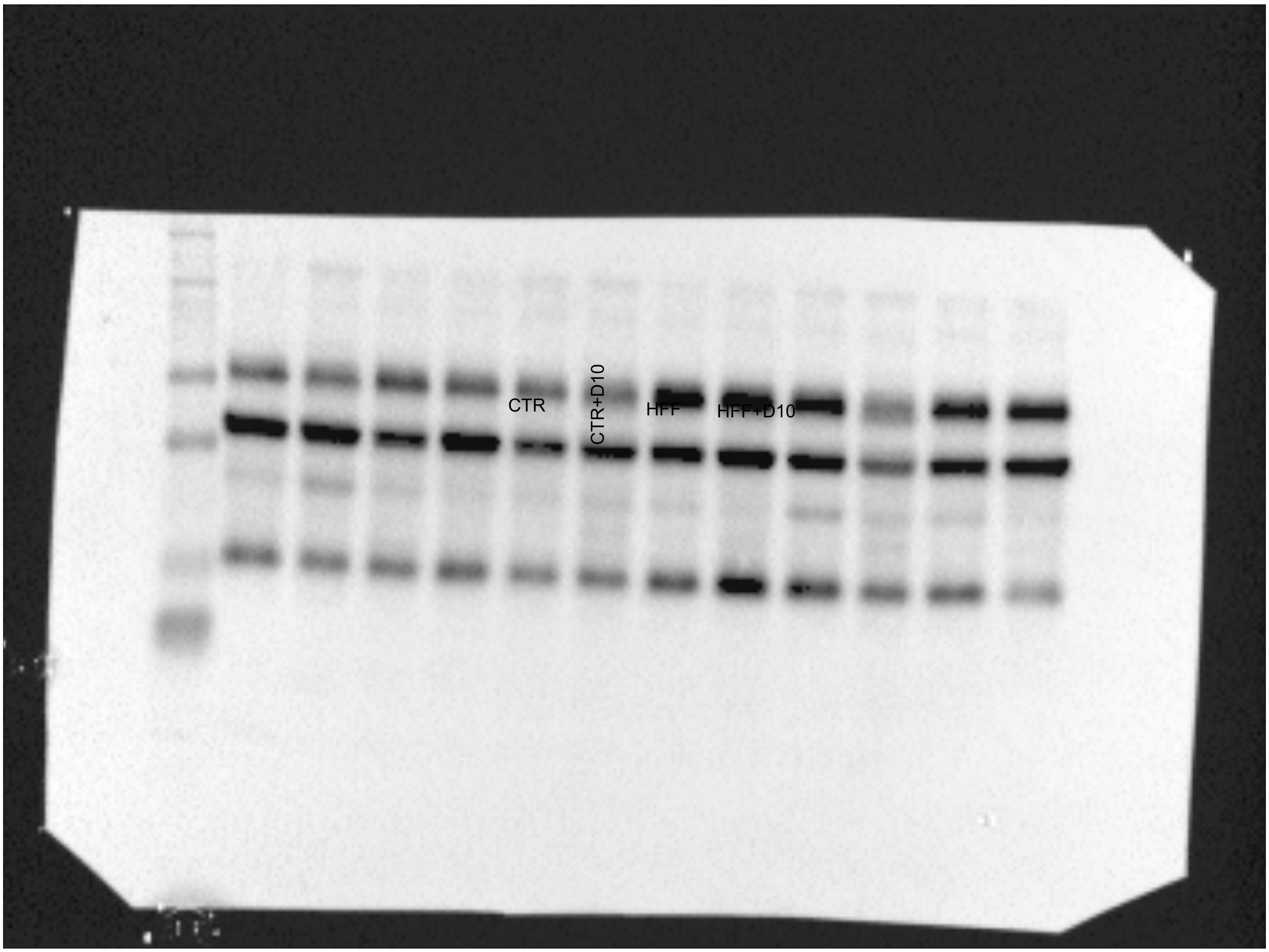

NRF1_membrana

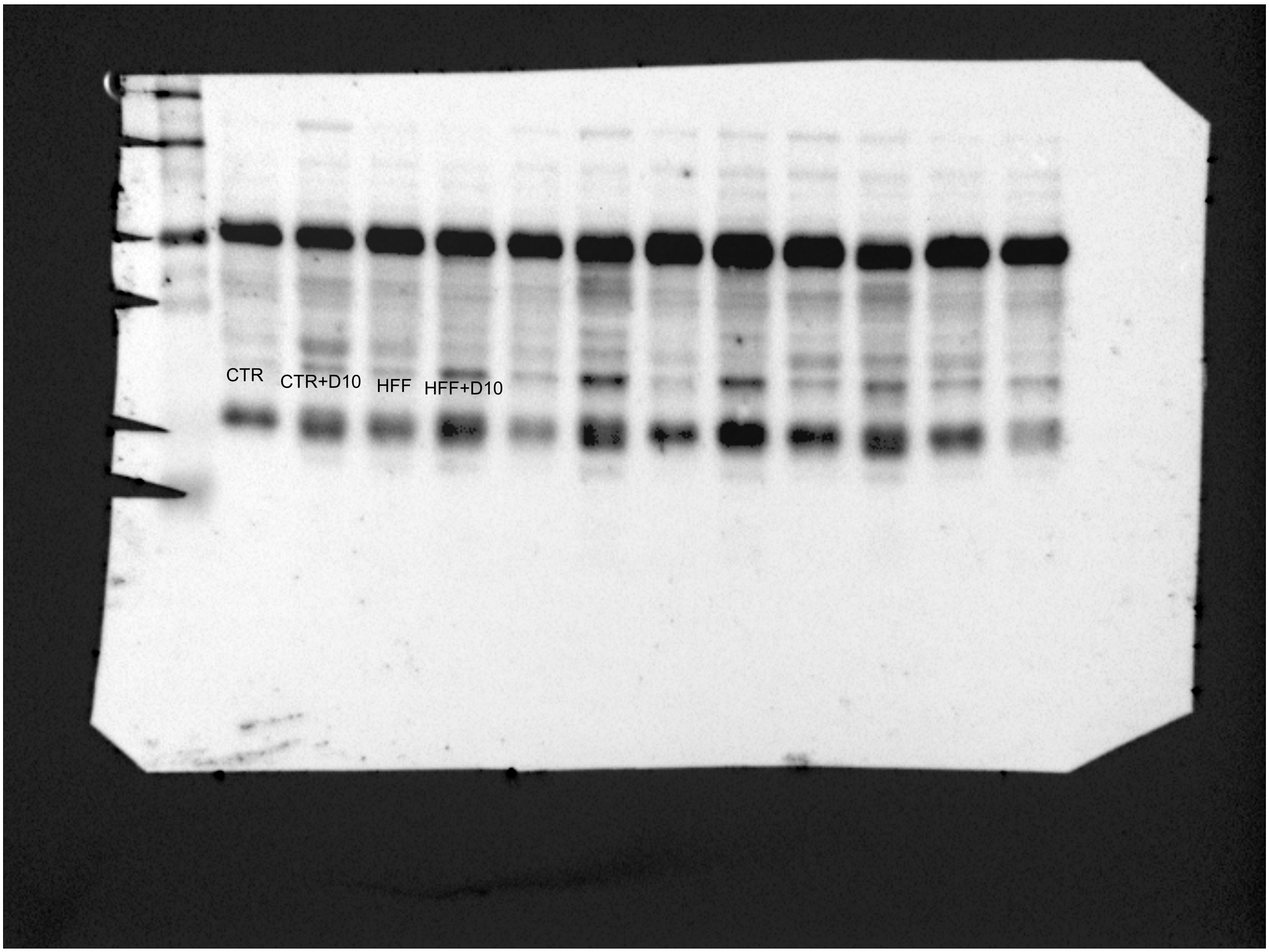

Membrana NT-PGC1a

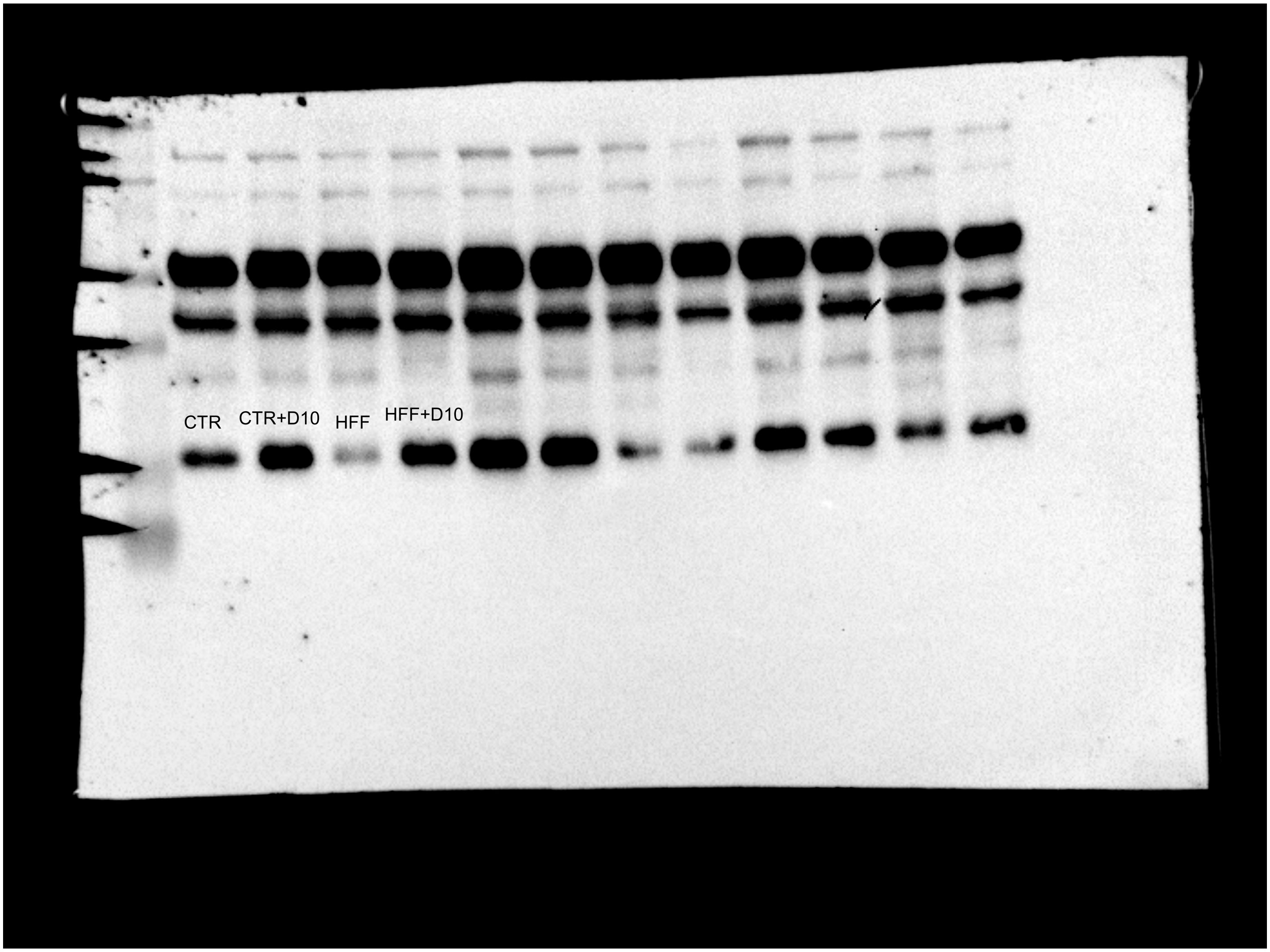

Membrana PGC1a_sec

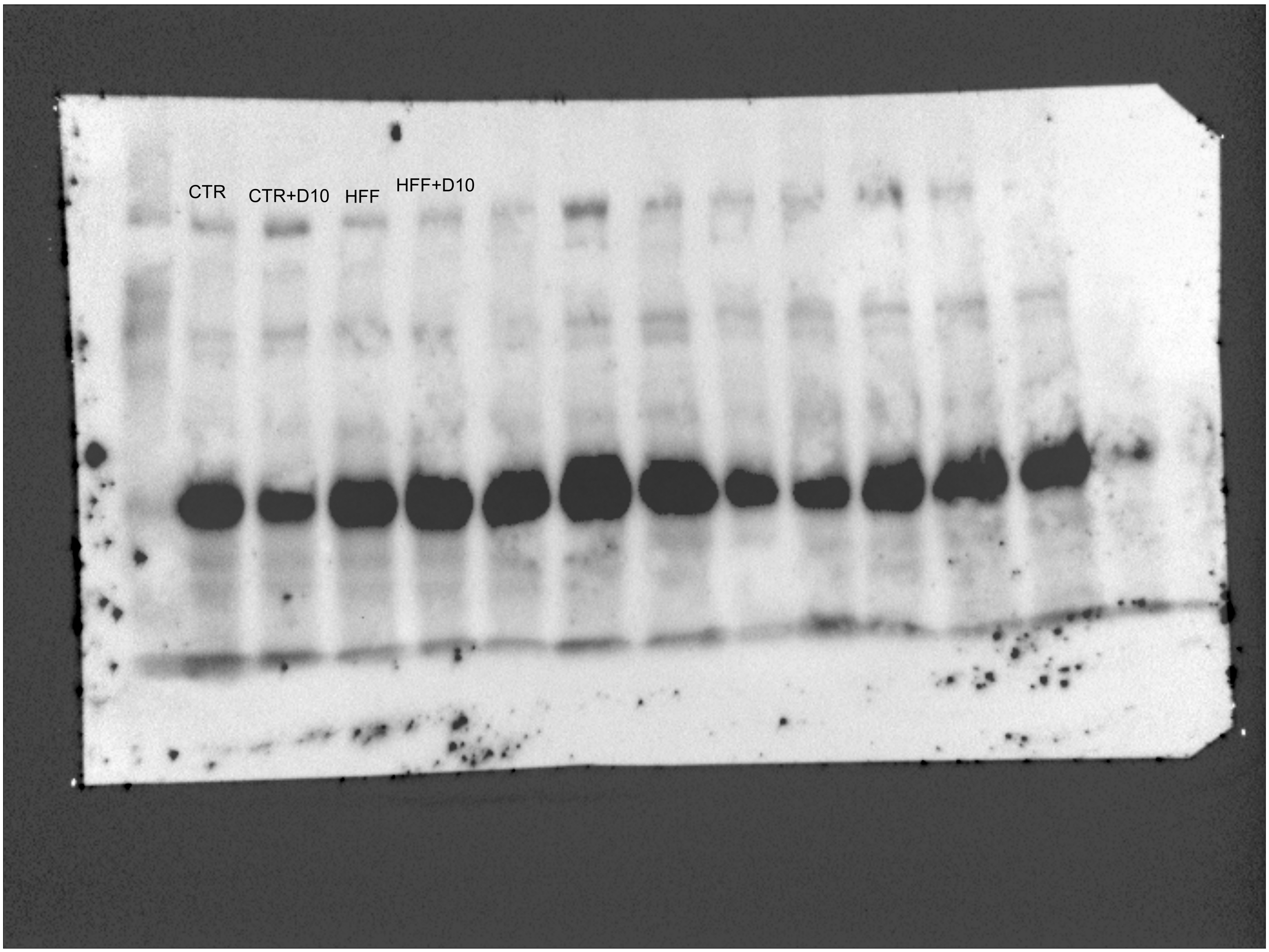

Membrana_SIRT1_Gel3

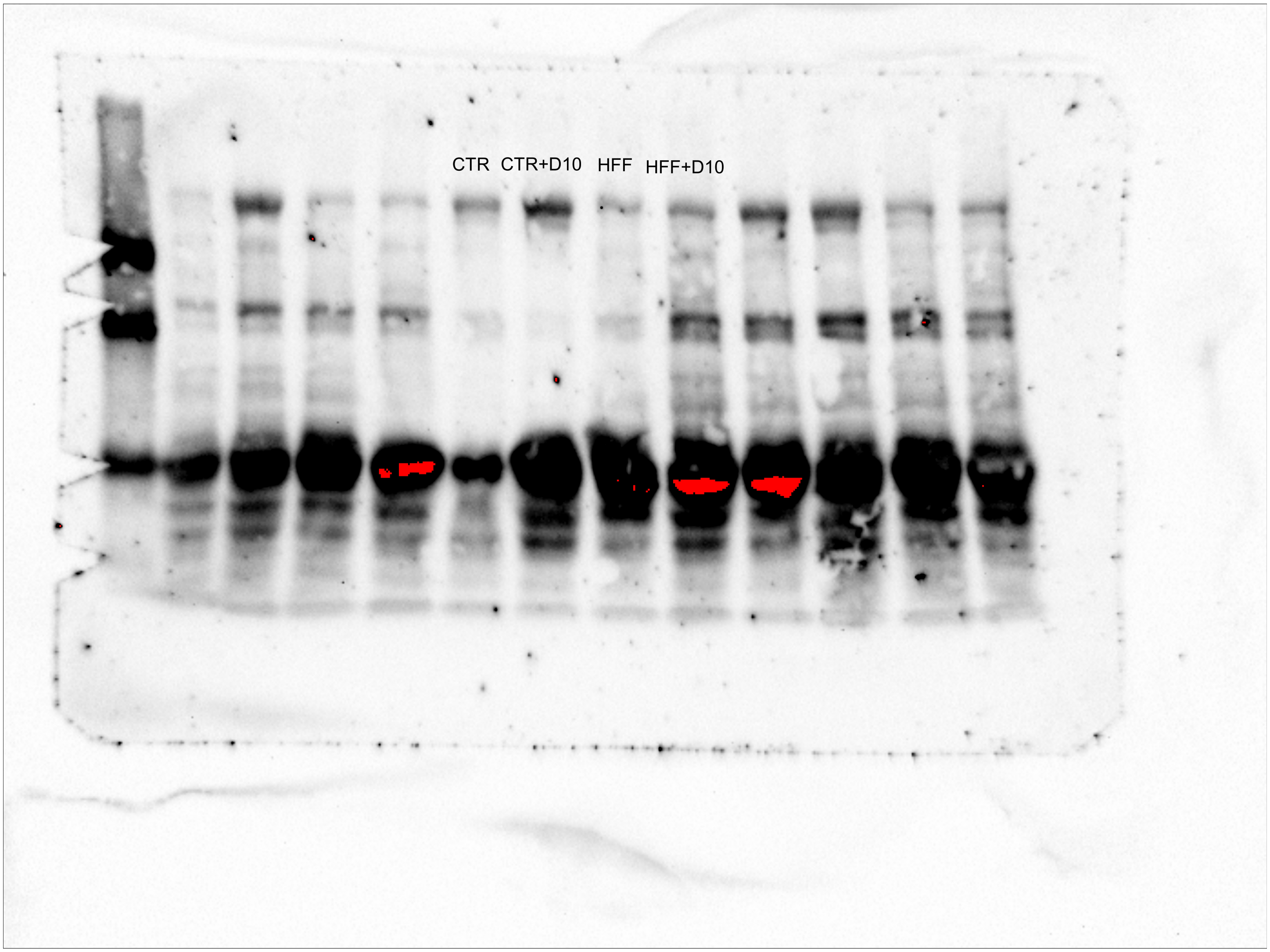

Supplement: Supplementary file 1 [file 210_2026_5723_MOESM1_ESM.pdf]
